# The impact of anemia on early postoperative complications in aseptic revision total shoulder arthroplasty

**DOI:** 10.1007/s00590-025-04372-8

**Published:** 2025-06-18

**Authors:** Steven H. Liu, Christian J. Leonardo, Rachel A. Loyst, Becka J. Konnayil, Allen Bramian, Edward D. Wang

**Affiliations:** 1 Department of Orthopaedic Surgery, Keck Medicine of University of Southern California, Los Angeles, United States; 2 Department of Orthopaedics, Stony Brook Medicine, Stony Brook, United States; 3 Department of Orthopaedic Surgery, University of California Los Angeles, Los Angeles, United States

**Keywords:** Revision total shoulder arthroplasty, Total shoulder arthroplasty, Hematocrit, Anemia, Complications

## Abstract

**Background:**

This study investigates the association between preoperative anemia and 30-day postoperative complications following aseptic revision total shoulder arthroplasty (TSA).

**Methods:**

The American College of Surgeons National Surgical Quality Improvement Program database was queried for all patients who underwent aseptic revision TSA from 2015 to 2022. The study population was divided into two cohorts based on preoperative hematocrit (HCT): normal (Male HCT ≥ 41%; Female HCT ≥ 36%) and anemia (Male HCT < 41%; Female HCT < 36%). Logistic regression analysis was conducted to investigate the relationship between anemia and postoperative complications following aseptic revision TSA.

**Results:**

Anemia was independently associated with any complication (OR 1.71, 95% CI 1.40–2.10; *P* < 0.001), blood transfusions (OR 8.71, 95% CI 4.23–17.90; *P* < 0.001), non-home discharge (OR 1.81, 95% CI 1.26–2.60; *P* = 0.001), and length of stay exceeding 2 days (OR 1.81, 95% CI 1.43–2.29; *P* < 0.001).

**Conclusion:**

Patients with anemia experienced significantly higher rates of early postoperative complications after aseptic revision TSA. These findings provide evidence for the use of preoperative HCT measurements as a practical predictor of postoperative risk in this setting.

**Level of Evidence** Level III; Retrospective Cohort Comparison; Prognosis Study.

## Introduction

Total shoulder arthroplasty (TSA) is becoming increasingly popular, with an increasing incidence year after year [1]. As a result, the incidence of revision TSA has also increased over four-fold from 2001 to 2010 [2]. As rates of both TSA and revision TSA continue to rise, along with an expanding demographic requiring surgical repair, physicians must carefully select optimal candidates by thoroughly evaluating preoperative criteria.

Surgeons routinely use preoperative laboratory values to help identify appropriate candidates for orthopedic surgery, and among these measures, hematocrit (HCT) is especially informative. A reduced HCT has been linked to worse surgical outcomes and a higher likelihood of perioperative blood transfusion [3–5]. Anemia, defined by a low HCT, affects up to 35% of patients undergoing orthopedic procedures and has been associated with multiple 30-day postoperative complications following TSA [6]. Multiple studies have consistently demonstrated that preoperative anemia is a risk factor for postoperative complications following total joint arthroplasty [7–9]. In addition, patients with severe anemia have been found to face significantly greater perioperative morbidity and mortality following non-cardiac surgery [10].

Although the role of anemia has previously been studied following various orthopedic surgeries [6, 11, 12], no studies have examined its role in the setting of revision TSA. The present study focuses on determining the relationship between preoperative anemia and 30-day postoperative complications following revision TSA. We hypothesized that preoperative anemia would be associated with higher rates of 30-day postoperative complications following aseptic revision TSA.

## Materials and methods

The American College of Surgeons National Surgical Quality Improvement Program (ACS-NSQIP) database was queried for all patients who underwent revision TSA from 2015 to 2022. This study was exempt from approval by our University’s Institutional Review Board because the NSQIP database is fully deidentified. Data in the NSQIP database are gathered from over 600 hospitals in the USA by trained surgical clinical reviewers. The data are periodically reviewed to maintain high reliability.

**Fig. 1:**
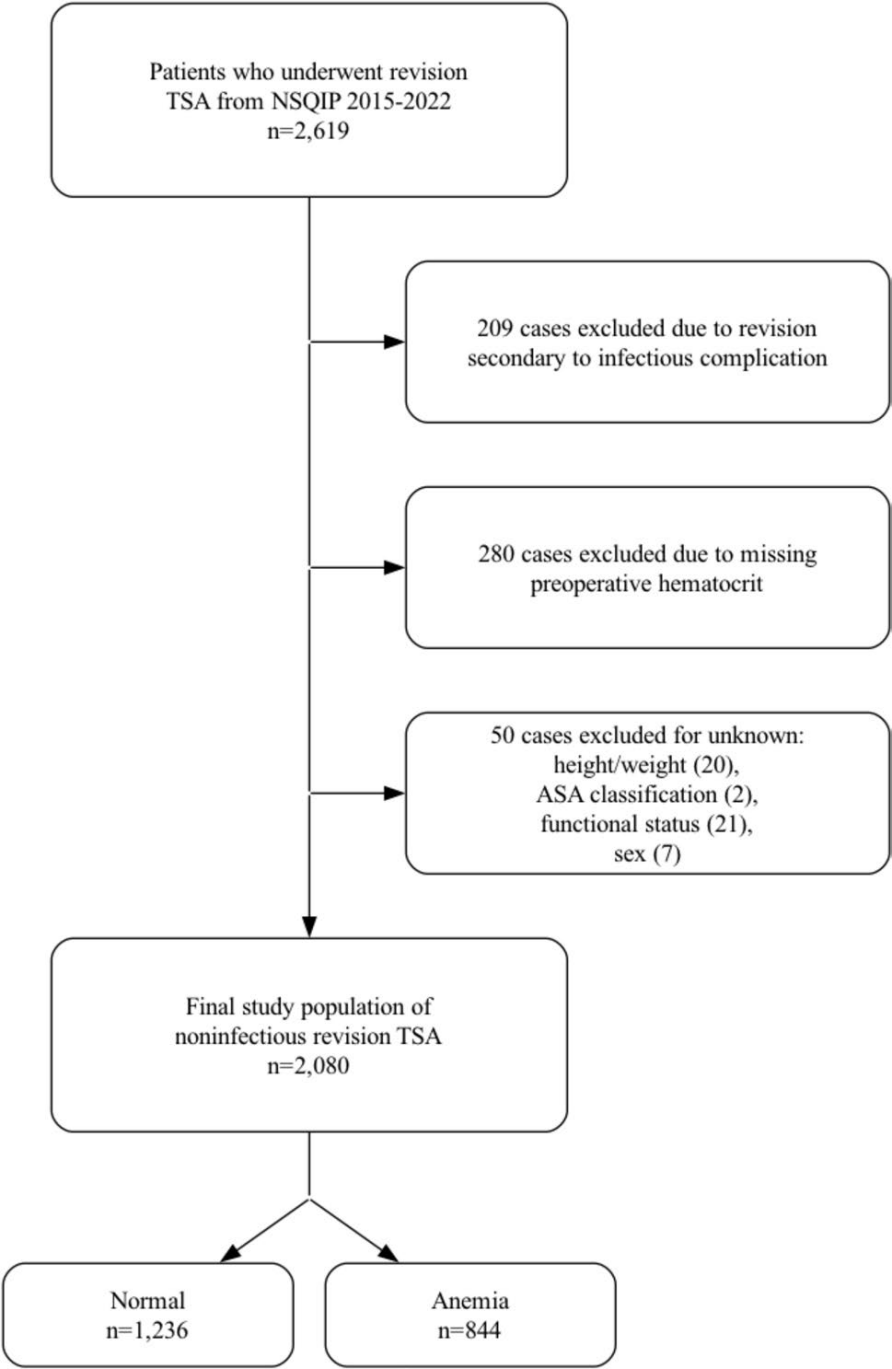
Case selection schematic. TSA, total shoulder arthroplasty; *NSQIP*, National Surgical Quality Improvement Program; ASA, American Society of Anesthesiologists

The *Current Procedural Terminology* (CPT) codes 23473 and 23474 were used to identify 2,619 patients who underwent revision TSA from 2015 to 2022 (Fig. 1). The NSQIP database inherently excludes all cases for patients younger than 18 years of age and all cases with primary admission related to trauma. Variables collected in this study included patient demographics, comorbidities, surgical characteristics, and 30-day postoperative complication data. Laboratory studies reported in the NSQIP database are obtained within a 30-day period prior to the procedure. Patient demographics included sex, age, body mass index (BMI), functional status, ASA classification, smoking status, and preoperative steroid use. Preoperative comorbidities included congestive heart failure (CHF), diabetes mellitus, hypertension, severe chronic obstructive pulmonary disease (COPD), bleeding disorders, and disseminated cancer. Perioperative factors included total operation time. Thirty-day complications included the following: sepsis, septic shock, pneumonia, unplanned reintubation, urinary tract infection (UTI), cardiac arrest or myocardial infarction (MI), stroke, blood transfusions, deep vein thrombosis (DVT), pulmonary embolism (PE), on ventilator > 48 h, surgical site infection (SSI), wound dehiscence, acute renal failure, *Clostridioides difficile*** (***C. diff*) infection, non-home discharge, readmission, unplanned reoperation, periprosthetic fracture, length of stay (LOS) > 2 days, and mortality.

Two hundred and nine revision TSA cases were excluded due to revision for an infectious etiology because the NSQIP database does not contain details regarding the nature of the infection (e.g., acute vs. chronic), which may impact complication rates. Next, 280 cases were excluded due to missing preoperative HCT measurements. Additionally, 50 cases were excluded for unknown height/weight, American Society of Anesthesiologists (ASA) classification, functional health status, or sex. After applying exclusion criteria, 2,080 cases remained and were stratified by preoperative HCT into non-anemic (male HCT ≥ 41%, female HCT ≥ 36%; *n* = 1,236) and anemic (male HCT < 41%, female HCT < 36%; *n* = 844) groups.

Statistical analyses were conducted in Python version 3.8 using the Statsmodels package. We first performed bivariate logistic regression to compare patient demographics and comorbidities between anemic and non-anemic groups. Variables with *P* < 0.05 in these bivariate analyses were entered as covariates in a multivariate logistic regression to identify independent associations between anemia and postoperative complications. Results are presented as odds ratios (OR) with 95% confidence intervals (CI), and statistical significance was defined as *P* < 0.05.

## Results

A total of 2080 patients met inclusion criteria (1236 non-anemic and 844 anemic). Compared with non-anemic patients, those with preoperative anemia were significantly older (15.3% vs. 8.7% ≥ 80 years; *P* < 0.001), more often had dependent functional status (6.0% vs. 1.9%; *P* < 0.001), and carried higher ASA classifications (73.8% vs. 58.0% ASA ≥ 3; *P* < 0.001). A greater proportion of anemic patients had a body mass index between 18.5 and 24.9 kg/m^2^ (18.7% vs. 14.6%; *P* = 0.025). They also exhibited a greater prevalence of diabetes (25.2% vs. 17.0%; *P* < 0.001), hypertension (74.9% vs. 62.1%; *P* < 0.001), and bleeding disorders (5.1% vs. 2.4%; *P* = 0.001). Fewer anemic patients were current smokers (8.8% vs. 12.4%; *P* = 0.010), while a greater proportion were on chronic steroids (7.3% vs. 5.2%; *P* = 0.043) (Table 1).

**Table 1:** Demographics and comorbidities of patients without anemia and patients with anemia

	Normal	Anemia	
Characteristics	Number (%)	Number (%)	*P* value
Overall	1,236 (100.0)	844 (100.0)	
Sex			0.728
Female	651 (52.7)	438 (51.9)	
Male	585 (47.3)	406 (48.1)	
Age			** < 0.001**
18–39	13 (1.1)	5 (0.6)	
40–59	217 (17.6)	117 (13.9)	
60–79	898 (72.7)	593 (70.3)	
≥ 80	108 (8.7)	129 (15.3)	
BMI (kg/m^2^)			**0.025**
< 18.5	6 (0.5)	4 (0.5)	
18.5–24.9	181 (14.6)	158 (18.7)	
25–29.9	373 (30.2)	251 (29.7)	
≥ 30	676 (54.7)	431 (51.1)	
Functional status prior to surgery			** < 0.001**
Dependent	24 (1.9)	51 (6.0)	
Independent	1,212 (98.1)	793 (94.0)	
ASA classification			** < 0.001**
≤ 2	519 (42.0)	221 (26.2)	
≥ 3	717 (58.0)	623 (73.8)	
Smoker			**0.010**
No	1083 (87.6)	770 (91.2)	
Yes	153 (12.4)	74 (8.8)	
Steroid use			**0.043**
No	1172 (94.8)	782 (92.7)	
Yes	64 (5.2)	62 (7.3)	
Comorbidities			
CHF	17 (1.4)	20 (2.4)	0.096
Diabetes	210 (17.0)	213 (25.2)	** < 0.001**
Hypertension	767 (62.1)	632 (74.9)	** < 0.001**
COPD	92 (7.4)	67 (7.9)	0.677
Bleeding disorder	30 (2.4)	43 (5.1)	**0.001**
Disseminated cancer	2 (0.2)	4 (0.5)	0.214
Total operation time (minutes)			0.263
0–79	263 (21.3)	202 (23.9)	
80–128	499 (40.4)	328 (38.9)	
≥ 129	474 (38.3)	314 (37.2)	

BMI, body mass index; ASA, American Society of Anesthesiologists; CHF, congestive heart failure; COPD, chronic obstructive pulmonary disease. Bold *P* Values indicate statistical significance with *P* < .05

Bivariate analysis of 30-day postoperative complications revealed that anemic patients experienced higher rates of any complication (35.1% vs. 21.3%; *P* < 0.001), blood transfusion (7.0% vs. 0.7%; *P* < 0.001), non-home discharge (11.0% vs. 4.6%; *P* < 0.001), and prolonged length of stay (> 2 days: 25.6% vs. 13.8%; *P* < 0.001) (Table 2).

**Table 2:** Bivariate analysis of 30-day postoperative complications in patients without anemia and patients with anemia

	Normal	Anemia	
Complications	Number (%)	Number (%)	*P* Value
Any complication	263 (21.3)	296 (35.1)	** < 0.001**
Sepsis	8 (0.6)	4 (0.5)	0.610
Septic shock	0 (0.0)	1 (0.1)	0.996
Pneumonia	1 (0.1)	4 (0.5)	0.113
Unplanned reintubation	1 (0.1)	3 (0.4)	0.199
UTI	8 (0.6)	7 (0.8)	0.631
Cardiac arrest or MI	2 (0.2)	6 (0.7)	0.069
Stroke	0 (0.0)	0 (0.0)	–
Blood transfusions	9 (0.7)	59 (7.0)	** < 0.001**
DVT	5 (0.4)	9 (1.1)	0.081
PE	6 (0.5)	6 (0.7)	0.508
On ventilator > 48 h	1 (0.1)	1 (0.1)	0.787
SSI	30 (2.4)	25 (3.0)	0.456
Wound dehiscence	4 (0.3)	0 (0.0)	1.000
Acute renal failure	2 (0.2)	1 (0.1)	0.799
Clostridioides difficile infection	1 (0.1)	1 (0.1)	0.787
Non-home discharge	57 (4.6)	93 (11.0)	** < 0.001**
Readmission	51 (4.1)	45 (5.3)	0.199
Unplanned reoperation	39 (3.2)	28 (3.3)	0.837
Periprosthetic fracture	0 (0.0)	0 (0.0)	–
LOS > 2 days	170 (13.8)	216 (25.6)	** < 0.001**
Mortality	1 (0.1)	1 (0.1)	0.787

UTI, urinary tract infection; MI, myocardial infarction; DVT, deep vein thrombosis; PE, pulmonary embolism; SSI, surgical site infection; LOS, length of stay. Bold *P* Values indicate statistical significance with *P* < .05

Multivariate logistic regression, adjusted for all significantly associated patient demographics and comorbidities, revealed that anemia remained independently associated with increased odds of any complication (OR 1.71; 95% CI, 1.40–2.10; *P* < 0.001), blood transfusion (OR 8.71; 95% CI, 4.23–17.90; *P* < 0.001), non-home discharge (OR 1.81; 95% CI, 1.26–2.60; *P* = 0.001), and LOS > 2 days (OR 1.81; 95% CI, 1.43–2.29; *P* < 0.001) (Table 3).

**Table 3:** Multivariate analysis of 30-day postoperative complications in patients without anemia and patients with anemia

Complications	OR, *P* value, (95% CI)
Any complication	1.71, < **0.001**, (1.40–2.10)
Blood transfusions	8.71, < **0.001**, (4.23–17.90)
Non-home discharge	1.81, **0.001**, (1.26–2.60)
LOS > 2 days	1.81, < **0.001**, (1.43–2.29)

LOS, length of stay

Bold *P* values indicate statistical significance with *P* < .05

## Discussion

Every year in the USA, the number of TSA procedures performed increases drastically [13–16]. A rise in the use of revision TSA procedures has also been observed, driven by the growing number of primary TSA procedures being performed [17–20]. Due to the increasing number of revision TSA procedures being performed each year, it is imperative that surgeons utilize preoperative factors, such as laboratory values, to assist in preoperative planning and risk stratification.

HCT is an example of a preoperative factor that can provide utility in identifying strong surgical candidates for revision TSA. Anemia is defined as a HCT level of less than 41% in men or less than 36% in women [21]. In our study, anemia was determined to be associated with various patient demographics and comorbidities, including older age, which is consistent with preexisting literature [22]. This study also provides evidence for anemia as a risk factor for postoperative complications following revision TSA, similar to what studies on primary TSA and other orthopedic procedures have shown [6–9]**.**

Anemia may be detected prior to surgery through routine laboratory testing and is a treatable condition. Previous research has demonstrated an association between preoperative anemia and adverse outcomes after both cardiac and non-cardiac surgery [23, 24]. Preoperative anemia is thought to be a surgical risk factor as it is associated with an increased risk of perioperative transfusion which increases the risk of morbidity and mortality [4, 5, 25, 26]. In addition, anemia may result in many complications including cerebrovascular events, non-home discharge, readmission, reoperation, and renal compromise [12, 27].

In the present study, we found anemia to be significantly associated with any complication, blood transfusions, non-home discharge, and LOS > 2 days in patients undergoing revision TSA. Similarly, previous studies have shown an increased length of hospital stay and reduced postoperative function among patients with anemia suffering from hip fractures [28–30]. Longer hospital stays and higher complication rates have also been observed in primary knee arthroplasty and in both primary and revision hip arthroplasty [31, 32]. Additionally, severe preoperative anemia was found to be a clinically significant predictor of postoperative complications such as higher rates of blood transfusion, non-home discharge, and mortality after open reduction and internal fixation in patients with distal radial fractures [33]. These findings highlight the importance of identifying anemia during the preoperative period to improve patient outcomes in revision TSA.

Building on this evidence, studies have explored the cost-effectiveness of treating anemia prior to orthopedic procedures. A cost‐simulation model demonstrated that preoperative anemia management in orthopedic patients both enhances clinical outcomes and reduces hospital expenditure [34]. The cost reduction reported was based on the assumption that patients who were screened and treated for iron-deficiency anemia would have similar outcomes to those without preoperative anemia [34]. This underscores the need for further research to determine the true clinical utility of screening for and treating preoperative anemia; however, if proven effective, such efforts may lead to better patient outcomes and reduced costs. This would not only enhance quality of care by reducing the risk of complications but also offers a financially advantageous strategy for healthcare systems, aligning with broader efforts to optimize both patient and economic outcomes in surgical settings. Specifically, identifying anemia during preoperative screening can help reduce adverse outcomes following revision TSA while lowering medical expenses.

There exist limitations to the present study, many of which are inherent to the use of a large database as in this study. Our study was limited to complications occurring within a 30-day postoperative period which precluded our ability to evaluate events that occurred beyond that time. Additionally, we were unable to account for the specific indications that necessitated revision TSA. As different indications for this procedure carry various degrees of risk, we were not able to account for this potentially confounding variable. Other unmeasured confounders could not be adjusted for, and reliance on a large database introduces the potential for coding inaccuracies. This database also lacks information on the procedure’s location and surgeon identity, preventing assessment of the surgical team’s experience. While we acknowledge these limitations, this study provides evidence for anemia as a significant risk factor for postoperative complications following revision total shoulder arthroplasty and underscores the need to develop more precise preoperative screening strategies to optimize patient outcomes.

## Conclusion

Following revision TSA, patients with anemia were independently associated with experiencing any complication, blood transfusion, non-home discharge, and LOS > 2 days when compared to patients without anemia. This study provides evidence for preoperative anemia as a risk factor for postoperative complications following revision total shoulder arthroplasty and may assist in preoperative risk stratification in this setting. Future studies investigating the utility of screening for anemia preoperatively may help to guide clinical care.

## Data Availability

No datasets were generated or analyzed during the current study.
